# Recent Clinical Characteristics of Labors Using Three Japanese Systems of Midwife-Led Primary Delivery Care

**DOI:** 10.1155/2016/9101479

**Published:** 2016-02-29

**Authors:** Shunji Suzuki

**Affiliations:** Department of Obstetrics and Gynecology, Japanese Red Cross Katsushika Maternity Hospital, Tokyo 124-0012, Japan

## Abstract

*Objective*. The objective of this study was to describe the recent clinical characteristics of labor using 3 systems of Japanese midwife-led primary delivery care, as follows: (1) those intending to give birth at home managed by midwives who do not belong to our hospital, (2) those planning to give birth in our hospital managed by the same midwives, and (3) those planning to give birth managed by midwives who belong to our hospital.* Methods*. A retrospective cohort study was performed.* Results*. There were no significant differences in the obstetric or neonatal outcomes among the 3 groups. The rate of transfers during labor with the system involving midwives belonging to our hospital was higher than those with the other 2 systems. In addition, the timing of transfers in the system with the midwives belonging to our hospital was earlier than with the other 2 systems. Among the 3 groups, there were no significant differences in the rate of the main 2 indications for transfers: fetal heart rate abnormality and failure to progress.* Conclusion*. There were no significant differences in perinatal outcomes among the 3 systems; however, there were some differences in the status of transfers to obstetric shared care.

## 1. Introduction

Midwife-led primary delivery care for “low-risk” pregnant women during labor has been reported to have various advantages, such as increased odds of high maternal satisfaction and a decrease of unnecessary medical interventions [1–8]. Although the maternity care system for “low-risk” pregnant women peculiar to one country cannot easily be compared with those in other countries, consumer demands for the “humanization” of obstetric care have arisen in various countries [1–8]. To date, we have found no evidence that midwife-led primary obstetric care is unsafe for “low-risk” pregnant women in comparison with obstetric care with the favorable cooperation of obstetricians and midwives in Japan [9–12]. In addition, about 85% of “low-risk” pregnant women request that they give birth while receiving midwife-led primary delivery care [10]. Therefore, safe midwife-led delivery care with the backup of obstetricians may also be required for “low-risk” pregnant women in Japan. If complications occur or threaten to occur during the primary midwife-led delivery care, the midwives have to refer the woman to obstetricians at the same or a neighboring hospital or private obstetric clinic as soon as possible. This is because, in deliveries managed by independent midwives in Japan, many intervention measures, such as oxytocin infusion, epidural anesthesia, episiotomy, suture, and instrumental delivery, are not available based on Japanese legal restrictions.

In our institute, one of the main Tokyo city perinatal centers, there are 3 Japanese systems of midwife-led delivery care, as follows: (1) those intending to give birth at home managed by midwives who do not belong to our hospital, (2) those planning to give birth on “futons” (i.e., Japanese-style bedding) in Japanese tatami mat delivery rooms in our hospital managed by the same midwives who do not belong to our hospital, and (3) those planning to give birth in Japanese tatami mat delivery rooms managed by midwives who belong to our hospital. The objective of this study was to describe trends in transfers and perinatal outcomes among labors using these 3 Japanese systems of midwife-led primary delivery care.

## 2. Methods

The protocol for this analysis was approved by the Ethics Committee of the Japanese Red Cross Katsushika Maternity Hospital. In addition, informed consent for analysis from a retrospective database was obtained from each subject during their hospital visit.

In our hospital, pregnant women who are initially considered “low-risk” at 34–36 weeks of gestation can choose freely between the 3 systems of midwife-led care and obstetric shared care. In the midwife-led care units, midwives can practice autonomously and are fully accountable for their own practice, unsupervised by obstetricians.

Factors used to exclude women from the “low-risk” group comprise the following [9–12]: (1) medical history: pregnancy-induced hypertension, chronic hypertension, diabetes mellitus, renal disease, idiopathic thrombocytopenia, and other systemic illnesses; (2) gynecological history: history of infertility therapies of in vitro fertilization, congenital uterine anomalies, uterine myomatosis, and adnexal anomaly; (3) obstetric history: narrowing of the pelvic outlet, cephalopelvic disproportion, previous Cesarean section, previous anal sphincter injury, previous postpartum hemorrhage ≥ 1,000 mL with blood transfusion, previous manual removal of placenta, previous gestational diabetes, and history of severe preeclampsia; (4) complications during the present pregnancy: multiple pregnancy, nonvertex presentation, obesity (maternal body mass index before pregnancy ≥ 25 and/or during the third trimester ≥ 28), anemia (hemoglobin < 9.0 g/dL), epilepsy with treatment, polyhydramnios, oligohydramnios, low-set placenta, placenta previa, fetal growth restriction, heavy for date fetus, gestational diabetes, and pregnancy-induced hypertension; when risk factors are present, those women are managed by obstetricians and midwives; (5) complications during labor: intrauterine infection, thick meconium staining, prolongation of labor such as active-phase dilation < 1 cm/hour and duration of second stage of labor ≥ 2 hours, prolonged rupture of membranes (≥24 hours), uterine inertia, arrest of labor, and fetal heart rate abnormality such as a nonreassuring fetal status. When these factors are present, the women are transferred to be managed mainly by obstetricians (obstetric shared care) in a standard Western-style delivery room or surgery room in our hospital.

A retrospective study was performed to examine trends in transfers and perinatal outcomes among labors that started using the 3 systems of midwife-led primary delivery care. In this study, neonatal asphyxia was defined as an Apgar score < 7 at 1 minute.

Student's* t*-test was used for continuous variables and the *χ* ^2^ test for categorical variables. Odds ratios (ORs) and 95% confidence intervals (CIs) were also calculated. Differences with *p* < 0.05 were considered significant.

## 3. Results

Between 2009 and 2012, a total of 678 low-risk women were placed in the 3 forms of midwife-led primary delivery care at the onset of labor pains and/or rupture of membranes at 37–41 weeks of gestation. Of these, 123 (18%) intended to give birth at home, 88 (13%) planned to give birth in the Japanese tatami mat rooms in our hospital managed by midwives who do not belong to our hospital, and 467 (59%) planned to give birth managed by the midwives belonging to our hospital.

Table 1 shows the clinical descriptions of the 678 pregnant women initially considered as “low-risk” for receiving our midwife-led primary delivery care systems. There were no significant differences in the maternal age or parity among the 3 groups.

Table 2 shows the rate of transfers in the 3 groups of the midwife-led primary delivery care systems. The total rate of transfers in the system run by the midwives belonging to our hospital (56%) was higher than in the other 2 systems run by the independent midwives (31% in planned home birth: OR 1.87, 95% CI 1.2–3.0, *p* < 0.01; 38% in planned hospital birth: OR 2.51, 95% CI 1.7–3.8, *p* < 0.01). In addition, the timing of transfers in the system run by the midwives belonging to our hospital (before the second stage of labor: 52%) was earlier than those in the other 2 systems (21% in the planned home birth: OR 4.12, 95% CI 2.6–6.6, *p* < 0.01; 20% in planned hospital birth: OR 4.29, 95% CI 2.5–7.4, *p* < 0.01). However, if classified into nulliparous and parous women, there were no significant differences in the rate of transfers among the 3 groups, as shown in Table 1. In addition, among the 3 groups there were no significant differences in the rate of the main 2 indications for transfer: fetal heart rate abnormality and failure to progress. The main indications for transfer after delivery were maternal postpartum hemorrhage and neonatal respiratory distress associated with asphyxia.

Table 3 shows the obstetric and neonatal outcomes in the pregnant women initially considered as “low-risk” for receiving our midwife-led primary delivery care systems. There were no significant differences in these outcomes among the 3 groups.

## 4. Discussion

Our obstetric care system involves the division of women in labor into low- and high-risk groups [9–12]. The women who are initially considered low-risk can choose freely between midwife-led care and obstetric shared care. If complications occur or risk factors arise during labor in the primary midwife-led care, they are transferred to obstetric shared care.

This may be the first report concerning the differences in the timing of transfers from midwife-led care to obstetric shared care among the 3 systems of midwife-led primary delivery care in Japan. In this study, there was no evidence that the primary midwife-led care is unsafe for “low-risk” pregnant women in any of these 3 midwife-led delivery care systems. The current results support some of our previous observations [9–12]. However, there were no significant differences in the timing of referrals from midwife-led care to obstetric shared care between the system led by midwives who belong to our hospital (hospital midwifery system) and the systems led by the midwives who do not belong to our hospital. In the hospital midwifery system, the timing of transfers seemed to be the earliest due to the ease of transfer within the same hospital and administrator setting. On the other hand, the rate of transfers after delivery with the other 2 systems was higher than that in the hospital midwifery care. During the period, the main indications for transfers were maternal postpartum hemorrhage and/or neonatal respiratory distress associated with asphyxia. Fortunately, the difference was not associated with adverse obstetric or neonatal outcomes; however, unfortunately, they led to early mother-to-child separation, especially in cases of planned home birth because healthy puerperal women or newborns cannot be transferred from home to hospital according to Japanese law. Although home birth might be very comfortable, those involved must be prepared for mother-to-child separation in cases of referrals after delivery.

The major limitations of this study were the small sample size and lack of long-term follow-up of mothers and children to consider the potential of the findings based on our own context. There were no cases of fetal/neonatal death under the midwife-led delivery care. The most evaluated outcome under midwife-led delivery was the satisfaction of pregnant women with the development of mother-child relationships after delivery. In addition, there might be some bias related to the backgrounds in the selection of the systems because this was not a randomized trial study. Therefore, a further large prospective study with long-term follow-up may be needed.

## 5. Conclusion

There are various systems of midwife-led primary delivery care in Japan. There were no significant differences in perinatal outcomes among the 3 systems; however, there were some differences in the status of the transfers to the obstetric shared care. Careful selection of the system may be needed.

## Figures and Tables

**Table 1:** Clinical descriptions of pregnant women initially considered as “low-risk” for receiving our 3 midwife-led primary delivery care systems.

Birth place	Planned home birth	Planned hospital birth	
Midwives	Independent midwives	Independent midwives	Midwives belonging to our hospital
Total number	123	88	467
Maternal age			
Average (years)	33.1 ± 4.9	33.2 ± 4.3	34.0 ± 4.8
≥35 years	39 (32%)	37 (42%)	215 (46%)
Nulliparity	32 (26%)	21 (24%)	154 (33%)

**Table 2:** Rate of transfers from midwife-led care to obstetric shared care in the 3 groups of the midwife-led primary delivery care systems.

Birth place	Planned home birth	Planned hospital birth	
Midwives	Independent midwives	Independent midwives	Midwives belonging to our hospital
Total number	123	88	467
Transfers			
Total	38 (31%)^*∗*^	33 (38%)^*∗*^	263 (56%)
Timing of transfers			
Before labor	12 (9.8%)	6 (6.8%)	40 (8.6%)
First & second stage of delivery	14 (11%)^*∗*^	12 (14%)^*∗*^	205 (44%)
Third stage of delivery	9 (7.3%)^*∗*^	13 (15%)^*∗*^	15 (3.2%)
Neonate only	3 (2.4%)^*∗*^	2 (2.3%)^*∗*^	3 (0.64%)
Nulliparous women			
Total	32	21	313
Transfers	17 (53%)	14 (67%)	219 (70%)
Multiparous women			
Total	91	67	154
Transfers	21 (23%)	19 (22%)	44 (27%)

^*∗*^ *p* < 0.05 versus the group managed by midwives belonging to the hospital (hospital midwifery care).

**Table 3:** Obstetric and neonatal outcomes in the pregnant women initially considered as “low-risk” for receiving our midwife-led primary delivery care systems.

Birth place	Planned home birth	Planned hospital birth	
Midwives	Independent midwives	Independent midwives	Midwives belonging to our hospital
Total number	123	88	467
Oxytocin use	16 (13%)	10 (11%)	92 (20%)
Instrumental delivery	3 (2.4%)	3 (3.4%)	23 (4.9%)
Cesarean delivery	2 (1.6%)	2 (2.4%)	6 (1.3%)
Neonatal birth weight			
Average (g)	3,165 ± 408	3,102 ± 351	3,131 ± 349
≥3,500 g	23 (19%)	10 (11%)	54 (12%)
Neonatal asphyxia	6 (4.9%)	4 (4.5%)	11 (2.4%)
Maternal blood loss ≥ 1,000 mL	8 (6.5%)	4 (4.5%)	19 (4.1%)
